# Magnetodielectric Effect in a Triangular Dysprosium Single‐Molecule Toroics

**DOI:** 10.1002/advs.202308220

**Published:** 2024-01-17

**Authors:** Yu‐Xia Wang, Yicheng Zhou, Yinina Ma, Peipei Lu, Yi‐Quan Zhang, Young Sun, Peng Cheng

**Affiliations:** ^1^ Tianjin Key Laboratory of Structure and Performance for Functional Molecules College of Chemistry Tianjin Normal University Tianjin 300387 P. R. China; ^2^ Key Laboratory of Advanced Energy Material Chemistry Frontiers Science Center for New Organic Matter and Haihe Laboratory of Sustainable Chemical Transformations (Tianjin) College of Chemistry Nankai University Tianjin 300071 P. R. China; ^3^ State Key Laboratory of Magnetism Institute of Physics Chinese Academy of Sciences Beijing 100190 P. R. China; ^4^ College of Physics Hebei Normal University Shijiazhuang 050024 China; ^5^ Jiangsu Key Lab for NSLSCS School of Physical Science and Technology Nanjing Normal University Nanjing 210023 P. R. China; ^6^ Center of Quantum Materials and Devices and Department of Applied Physics Chongqing University Chongqing 401331 P. R. China

**Keywords:** magnetodielectric effect, single‐molecule magnets, single‐molecule toroics, triangular dysprosium SMMs

## Abstract

Single‐molecule toroics are molecular magnets with vortex distribution of magnetic moments. The coupling between magnetic and electric properties such as the magnetodielectric effect will provide potential applications for them. Herein, the observation of significant magnetodielectric effect in a triangular Dy3 crystal with toroidal magnetic moment and multiple magnetic relaxations is reported. The analysis of magnetic and electric properties implies that the magnetodielectric effect is closely related to the strong spin‐lattice coupling, magnetic interactions of Dy^3+^ ions, as well as molecular packing models.

## Graphical abstract

Single‐molecule toroics are special molecular magnets with vortex distribution of magnetic moments and regarded as one of the magnetoelectric moments. Herein, a triangular Dy3 single‐molecule toroic exhibits clear negative magnetodielectric effects which indicates that larger magnetodielectric effects can be obtained in multinuclear magnetic clusters. This work brings an emerging new route to molecular magnetoelectrics.

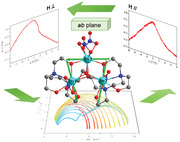

## 1 Introduction

Single‐molecule magnets (SMMs), a special class of molecule‐based magnets in which nanomagnetic units are isolated by organic ligands, have drawn widespread attention for their potential applications in high‐density spintronic devices^[^ ^1^ ^]^ and quantum computing^[^ ^2^, ^3^ ^]^ in the past two decades. SMMs are characterized by slow magnetic relaxation below a magnetic blocking temperature and unique resonant quantum tunneling of magnetization (RQTM)^[^ ^4^ ^]^ at low temperatures. Therein, SMMs with a vortex distribution of magnetic dipoles have received special attention and named single‐molecule toroics (SMTs).^[^ ^5^ ^]^ Among all SMTs, the triangular Dy3 cluster is an unique archetype system with peculiar magnetic behaviors.^[^ ^6^, ^7^, ^8^, ^9^, ^10^, ^11^, ^12^ ^]^ For example, the triangular Dy3 complex shows an abnormal nonmagnetic ground state due to the circular arrangement of magnetic moments of three Dy^3+^ ions.^[^ ^6^ ^]^ The toroidal magnetic moment is also regarded as a magnetoelectric moment in addition to the traditional polarization and magnetization. In our prior study, an asymmetric isosceles Dy3 SMM exhibited room‐temperature ferroelectricity arising from ethanol solvents but without the coupling between electric and magnetic properties.^[^ ^13^ ^]^

In strong contrast to the well‐studied magnetism, the electrical properties of SMMs including SMTs have been less investigated because these materials are usually good insulators.^[^ ^14^, ^15^ ^]^ The conventional electrical properties such as magnetoresistance and Hall effect are inapplicable for insulating SMMs. Instead, the dielectric properties of SMMs are measurable. Especially, the magnetodielectric (MD) effect, that is, the magnitude of dielectric constant is controlled by external magnetic field, has a potential for applications in low‐energy multifunctional devices. At present, the investigations are mainly focused on inorganic oxides, such as perovskite‐type BiFeO_3_ and BiMnO_3_, hexagonal RMnO_3_ (R = rare earth elements) and YbFeO_3_, polar polymorphs of ScFeO_3_, while only few studies were concentrated on molecular magnets.^[^ ^16^, ^17^, ^18^, ^19^, ^20^, ^21^ ^]^ In conventional magnetoelectric materials, ferroelectricity and ferromagnetism naturally coexist within the same phase. Bridging magnetodielectric properties in the unique SMTs would enrich the physical properties of SMMs and broaden the scope of magnetodielectric materials.

The MD effect of the Dy‐SMM arising from the strong spin‐lattice coupling of the Dy^3+^ ions.^[^ ^18^ ^]^ Hence, due to the unique vortex magnetic moments of SMTs and the magnetic interactions between Dy^3+^ ions, Dy3 clusters could exhibit magnetoelectric coupling in principle. In this work, we report the observation of toridal magnetic moment, multiple relaxations, and MD effect in an isosceles Dy3 cluster, [Dy_3_(HL)(H_2_L)(NO_3_)_4_] (**Dy3**, H_4_L = *N,N,N*’,*N*’‐tetrakis(2‐hydroxyethyl)ethylenediamine).

## 2 Results and Discussion

**Figure 1:**
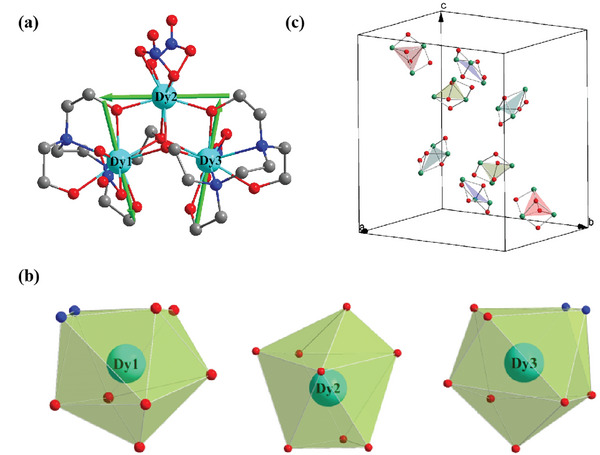
Crystalline structure and toroidal magnetic moments. Molecular structures of **Dy3** at 126 K. a) molecular structures of Dy3, the green axes represent the calculated orientations of the local main magnetic axes directions. b) Coordination geometries for Dy^3+^ ions. c) Dy3 molecule packing in a unit cell. Color scheme: green Dy, red O, gray C, blue N. The hydrogen atoms are omitted for clarity.

Millimeter‐sized single‐crystals of **Dy3** were obtained via solvent thermal method by a well‐controlled synthetic process. The high thermal stability and purity were confirmed by thermogravimetric analysis and powder X‐ray diffraction at 126 and 30 K(Figures S1 and S2; Table S1, Supporting Information). Single‐crystal X‐ray diffraction studies reveals that complex **Dy3** crystallizes in the orthorhombic space group *P*bca (**Figure** **1**). There are three crystallographic independent Dy^3+^ ions bridged by two *µ*_3_‐O (O1, O2) and two *µ*_2_‐O (O3, O4) atoms from two ligands (Figure 1a), respectively. Therefore, three Dy^3+^ ions show two types of coordination geometries, Dy1(Dy3) is in a distorted nine‐coordinated spherical capped square antiprism bonding to four O atoms and two N atoms from the HL^3−^ ligand, one O atom from the HL^2−^ ligand and two O atoms from one NO_3_^−^, while Dy2 adopts a distorted eight‐coordinated square antiprism geometry, which is completed by one O atom from the HL^3−^ ligand, three O atoms from the H_2_L^2−^ ligand and four O atoms from two NO_3_^−^ groups (Figure 1b).

Additionally, the continuous shape measure (CSM)^[^ ^22^ ^]^ calculations (Table S2, Supporting Information) reveal that Dy2 is situated in a slightly distorted *D*_4d_ geometry while Dy1 and Dy3 are situated within a *C*_4v_ coordination environment with different deviation values. There are eight triangular clusters in each unit cell that can be divided into four types (Figure 1c), which is different from the previous ferroelectric Dy3 complex (Figures S3 and S4, Supporting Information).^[^ ^12^, ^13^ ^]^ Different molecule packing models maybe generate different magnetic and electric behaviors.

**Figure 2:**
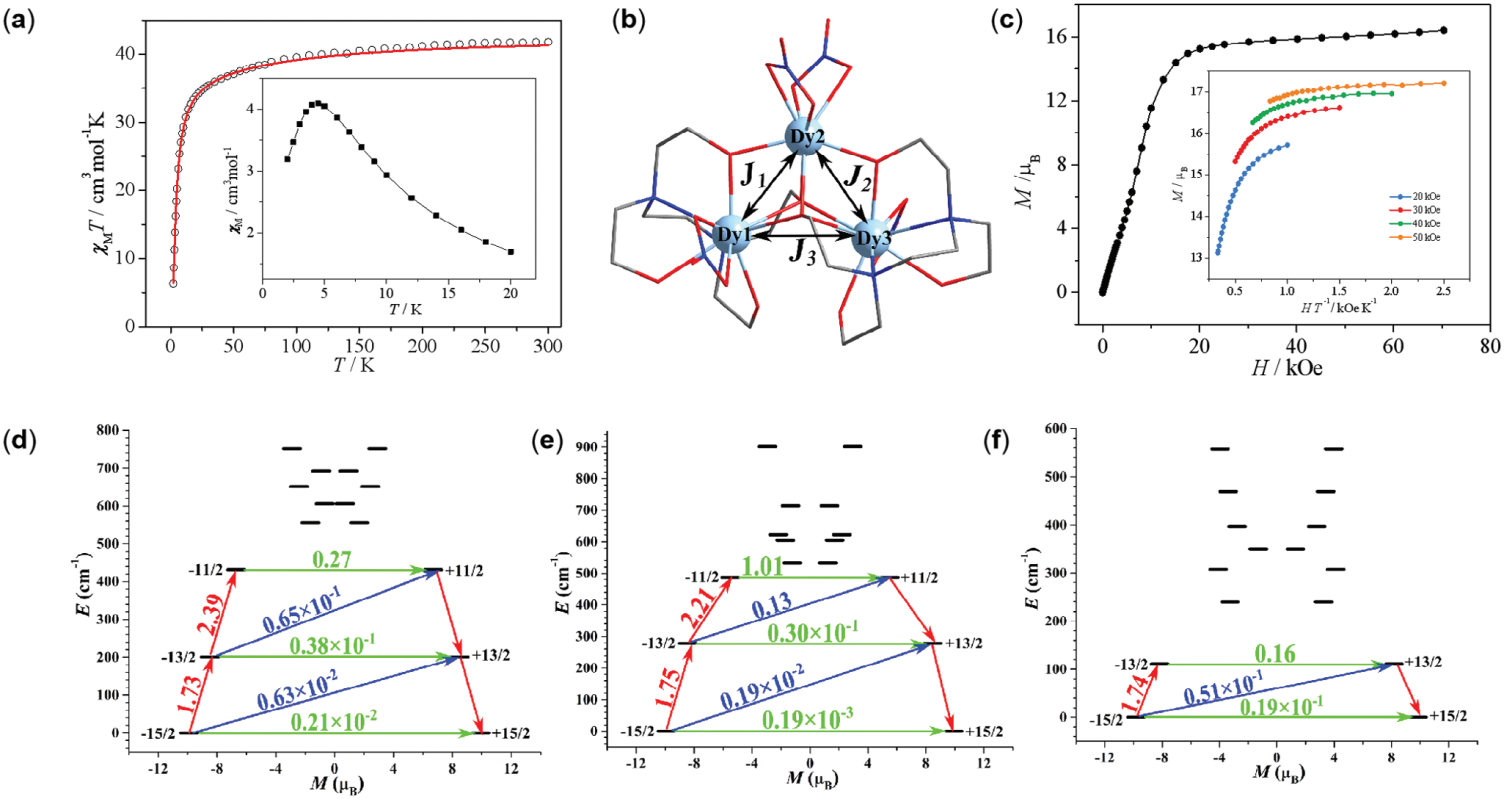
Magnetism analysis. a) Calculated (red solid line) and experimental (black circle) data of magnetic susceptibilities of **Dy3**. b) Scheme of the Dy^3+^−Dy^3+^ interactions in **Dy3**. The intermolecular interaction *zJ*´ of complex **Dy3** was fitted to −0.02 cm^−1^. c) *M*−*H* for **Dy3**, solid lines are for eye guide. d–f) Magnetization blocking barriers of individual Dy^3+^ fragments Dy1, Dy2 and Dy3 for **Dy3**, respectively. The thick black lines represent the Kramers doublets (KDs) as a function of their magnetic moment along the magnetic axis. The green lines correspond to diagonal quantum tunneling of magnetization (QTM); the blue line represent off‐diagonal relaxation process. The numbers at each arrow stand for the mean absolute value of the corresponding matrix element of transition magnetic moment.

Direct‐current (DC) magnetic susceptibility of **Dy3** (**Figure** **2**) were measured in the temperature range of 2—300 K under an applied magnetic field of 1 kOe. The *χ*_M_ *T* value at 300 K is 41.80 cm^3^ K mol^−1^, which is in good agreement with the expected value for three free Dy^3+^ ion (^6^ *H*_15/2_, *S* = 5/2, *L* = 5, *J* = 15/2, *g* = 4/3). The *χ*_M_ *T*–*T* curve gradually decreases upon lowering the temperature and reaches a minimum value of 6.37 cm^3^ K mol^−1^ at 2 K (Figure 2a). The decline of *χ*_M_ *T* can be ascribed to the progressive depopulation of the *M*_J_ states of the Dy^3+^ ions and/or intermolecular interactions. Besides, the molar magnetic susceptibility indicates an antiferromagnetic interaction as commonly observed in Dy3 SMMs (Figure 2a, inset).^[^ ^6^, ^12^, ^13^ ^]^ The field dependent magnetization for **Dy3** was illustrated in Figure 2c, the value of 15.84 *µ*_B_ (7 T, 2 K) is close to three uncorrelated Dy^3+^ magnetic moments (15.69 *µ*_B_), but far lower than the expected saturation values of 30 *µ*_B_. This may be due to crystal‐field effects/low‐lying excited states and eliminates the 16‐fold degeneracy of the ^6^H_15/2_ ground state. The plot of *M* versus *H*/*T* shown a non‐superimposition suggest the presence of the magnetic anisotropy and/or the lack of a well‐defined ground state, where the low‐lying excited states might be populated when a field is applied (Figure 2c, inset).^[^ ^23^ ^]^

Furthermore, complete‐active‐space self‐consistent field (CASSCF) calculations on three types of Dy^3+^ fragments (Figure S5, Supporting Information) for complex **Dy3** on the basis of X‐ray determined geometry have been carried out with the OpenMolcas^[^ ^24^ ^]^ and SINGLE_ANISO^[^ ^25^, ^26^, ^27^ ^]^ programs. The exchange coupling constants of $\tilde{J}$_exch_ and the intermolecular interaction *zJ*´ were fitted through comparison of the computed and measured magnetic susceptibilities for **Dy3**. The total coupling parameters $\tilde{J}$_total_ (dipolar and exchange) are included into fitting the magnetic susceptibilities. The calculated *χ*_M_ *T* versus *T* plot of complex **Dy3** is close to the experiment at low temperatures (Figure 2a).^[^ ^28^ ^]^ From Table S3 (Supporting Information), the *J*_1_, *J*_2_ and *J*_3_ in **Dy3** within lines model^[^ ^29^ ^]^ are all antiferromagnetic, and the intermolecular interaction *zJ*´ is −0.02 cm^−1^. We gave the exchange energies (cm^−1^), the transversal magnetic moments Δ*τ* (*µ*_B_) and the main values of the *g_z_* for the lowest four exchange doublets of **Dy3** (see Table S4, Supporting Information). The *g*_z_ value of the ground exchange states of **Dy3** is 11.689, which confirms that the Dy^3+^‐Dy^3+^ interactions in **Dy3** are all antiferromagnetic and in accordance with the obtained experimental results.

The calculated energy levels (cm^−1^), ***g*** (*g_x_*, *g_y_*, *g_z_*) tensors and the predominant *m_J_* values of the lowest eight Kramers doublets (KDs) of individual Dy^3+^ fragments for **Dy3** are shown in Table S5 (Supporting Information). The predominant *m_J_* components for the lowest eight KDs of individual Dy^3+^ fragments for **Dy3** are shown in Table S6 (Supporting Information), where the ground and the first excited KDs are mostly composed by *m_J_* = ±15/2 and *m_J_* = ±13/2, respectively. The second KD of each Dy^3+^ ion is composed of several *m*_J_ states, which leads to a larger transverse magnetic moments in the second excited KD.

The corresponding magnetization blocking barriers of individual Dy^3+^ fragments for complex **Dy3** are shown in Figure 2d–f, where the transversal magnetic moments in the ground KDs of them are all close to 10^−2^ *µ*_B_, and thus the quantum tunneling of magnetizations (QTMs) in their ground KDs are all suppressed at low temperatures. The transversal magnetic moments in their first excited KDs of individual Dy^3+^ fragments for complex **Dy3** are all small enough (ca. close to 0.30×10^−1^ *µ*_B_) to enable the relaxation to proceed through the second excited KDs except value (ca. 0.16 *µ*_B_). Thus, DC magnetic measurements and theory calculations imply that molecules packing would affect magnetic relaxations and magnetic anistropy as well.

**Figure 3:**
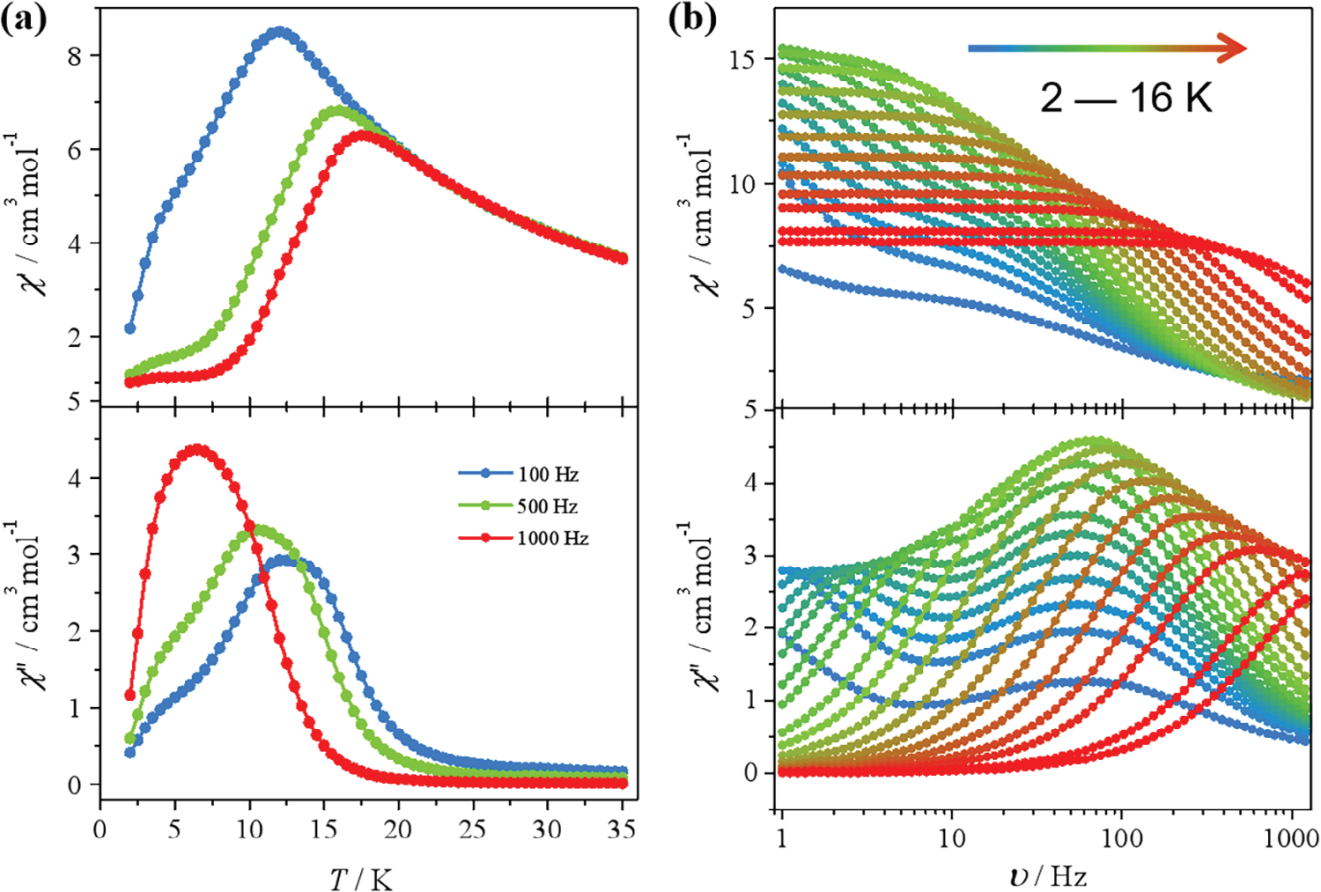
Magnetic relaxations. Temperature a) and frequency b) dependence of in‐phase (*χ*') and out‐of‐phase (*χ*””) components of AC magnetic susceptibility for the powder sample of **Dy3** under zero‐dc field.

Then, magnetization dynamics, one of the most important characteristics of SMMs, is investigated on the powder sample. Temperature and frequency dependence of alternating‐current (AC) magnetic susceptibilities show strong frequency dependence with increasing frequency and decreasing temperature (**Figure** **3**). As illustrated in the *χ“*(*v*) of **Dy3**, two obviously separate relaxation processes appear at high‐frequency (faster relaxation phase, FR) and low‐frequency (slower relaxation phase, SR), respectively. The different magnetic relaxation dynamics between **Dy3** and the ferroelectric Dy3 furthure confirmed that the molecular packing is a crucial influnce factor for spin‐lattice coupling. The Cole‐Cole plots of **Dy3** go through an evolution at the frequency range of the test corresponding to *χ”*(*v*) data and provide a much more indicative view of the above transformation. Two parts semicircles of the Cole‐Cole plots could be well described by the sum of two modified Debye functions on the entire processes,^[^ ^30^ ^]^

(1) $$\begin{array}{ccc} \chi_{ac}\left(\omega \right) & = & \chi_{s}+\sum_{k}\frac{\Delta \chi_{k}}{1+{\left(i\omega \tau \right)}^{\left(1-a_{k}\right)}},\;\chi_{s}\left(\omega \right)\\ & = & \sum_{k}\chi_{s,k};\;\Delta \chi_{k}=\chi_{T,k}-\chi_{s,k} \end{array}$$

where *χ*_s_ = *χ*_s1_ + *χ*_s2_ represents the sum of the adiabatic susceptibility of two relaxing species; Δ*χ*_κ_ is the difference between the adiabatic susceptibility (*χ*_s,κ_) and the isothermal susceptibility (*χ_T_*_,κ_) of each magnetic phase; *ω* = 2π*v* and the parameter *v* is the width of the relaxation time (*τ*) distribution.

**Figure 4:**
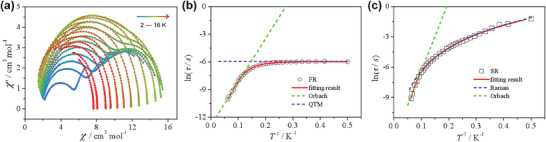
Magnetic relaxation dynamics. a) The Cole‐Cole plots of **Dy3**. The red lines represent the best fitting results. b) and c) are the best fitting results of the correlation between relaxation time (*τ*) and temperature (*T*) for the FR and SR, respectively.

The obtained small parameters *α*_1_ and *α*_2_ (*α* < 0.27) illustrate slight deviations from pure Debye processes. The relaxation times of **Dy3** were extracted from two ridges of the frequency‐dependent data by fitting the *χ'*(*v*) and *χ″*(*v*) curves between 2—16 K (Figures S6 and Table S6, Supporting Information). The best fit to piecewise power law equations show that QTM and Orbach relaxations dominated the FR process, while the SR process are dominated by Raman and Orbach relaxations (Figure S7, Supporting Information). The correlation between the relaxation time (*τ*) and temperature (*T*) can be obtained by fitting the plots of ln(*τ*) versus ln(*T*) with *τ* ^−1^ = *τ*_QTM_^−1^ + *τ*_0_^−1^exp(−*U*_eff_/*k*_B_ *T*) + *CT* ^n^ (*τ*_0_ is the preexponential factor, *U*_eff_ is the effective energy barrier, *C* and *n* are constant parameter values of the Raman process that do not have a physical basis, and *k*_B_ is the Boltzmann constant) over the whole temperature range (**Figure** **4**). The effective energy barrier (*U*_eff_) and pre‐exponential factor (*τ*_0_) are *U*_eff_ = 49.1(6) K, *τ*_0_ = 2.3(1) × 10^−6^ s with *τ*_QTM_^−1^ = 0.00233 s for FR, and *U*_eff_ = 112(9) K, *τ*_0_ = 1.4(9) × 10^−7^ s with *C* = 0.3(3) s^−1^ K and *n* = 3.2 for SR, respectively.

In order to better understand the magnetization dynamics, the calculated and measured results are compared and analyzed. For **Dy3**, the local magnetization axes on Dy^3+^ ions are almost on the triangular dysprosium plane. Thus, we gave the angles between the main magnetic axes on Dy^3+^ ions in their ground KDs and those between the magnetic axes and the triangular dysprosium plane (Table S7, Supporting Information), which indicates that the magnetic axes at the dysprosium sites for **Dy3** do not form a perfectly toroidal one. As for triangular dysprosium compounds with the perfectly toroidal magnetic moment, *χ*_M_ *T* almost vanishes at low temperature and reorientation of the toroidal magnetization requires consecutive transitions through three excited Kramers doublets of triangular dysprosium whose excitation energies represent the barrier of blockage of this magnetization. For our studied system, however, it has no perfectly toroidal magnetic moment and the energy barrier cannot be represented by the excitation energies. Thus, the energy barrier of complex **Dy3** is mainly determined by individual Dy^3+^ fragments. Due to the unfavorable effects of anharmonic phonons, Raman magnetic relaxation, QTM, et. al. on energy barrier, the experimental effective energy barrier *U*_eff_ is usually smaller than the calculated energy barriers of individual Dy^3+^ fragments.^[^ ^31^, ^32^, ^33^ ^]^

**Figure 5:**
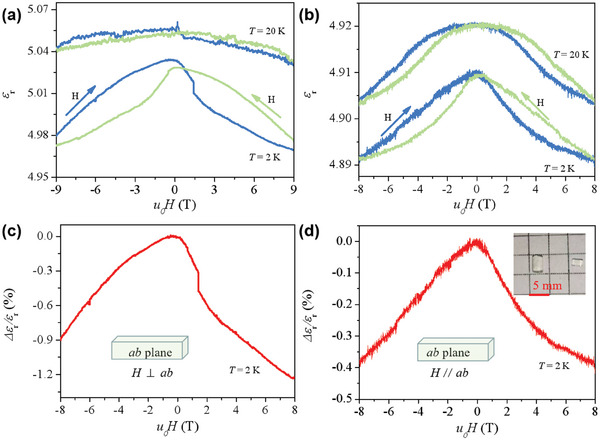
Magnetodielectric effects. Dielectric permittivity of **Dy3** as a function of applied magnetic field at temperatures of 2 and 20 K for a) *H* ⊥ *ab* plane and b) *H* // *ab* plane. The blue and green arrows indicate the ascending magnetic field and descending magnetic field. Relative change in the dielectric permittivity of **Dy3** as a function of applied ascending magnetic field at 2 K for c) *H* ⊥ *ab* plane and *H* // *ab* plane d).

To explore the MD effect, the *ε*_r_ value as a function of the magnetic field along different crystalline axes were measured on a single‐crystal sample (2×1×1 mm) at 2 and 20 K. For convenience, the larger surface (00$\bar{1}$) is denoted as the *ab* plane and chosen for painting electrodes (Figure S8, Supporting Information). Consequently, the electric field is oriented perpendicular to the ab plane. As shown in **Figure** **5**a,b, the *ε*_r_ values for all temperatures decreases with increasing magnetic field in the high‐field region, showing a negative MD effect. The calculated relative changes in dielectric permittivity (Δ*ε*/*ε*(0 T), where Δ*ε* = *ε*(*H*) – *ε*(0 T)) are shown in Figure 5c,d. Below magnetic blocking temperature, the magnitude of Δ*ε*/*ε*(0 T) for *H* ⊥ *ab* plane is smaller than that for *H* // *ab* plane, exhibiting anisotropic behavior which is consistent with the magnetic anisotropy (Figure S9, Supporting Information). The absolute maximum value of Δ*ε*/*ε*(0 T) for *H* ⊥ *ab* plane is ≈1.2% and unsaturated up to 8 T, which means that a higher Δ*ε*/*ε*(0 T) value would be reached under a higher magnetic field.

To gain further insight into the factors of MD effect in SMMs, relative dielectric permittivity changes as applied external magnetic fields are analyzed between **Dy3** and the previous mononuclear Dy‐SMM.^[^ ^18^ ^]^ Since the crystal‐space group of **Dy3** is non‐polar *P*bca at 30 K, and no dielectric anomaly was observed over the temperature range of 2—300 K (Figure S10, Supporting Information), thus this SMM persists the same symmetry persisted down to 2 K and cannot exhibit ferroelectricity in theory. Herein, both crystals are non‐ferroelectric SMMs but exhibit prominent MD effect. From a microscopic origin view, the crystal lattice can be modified by the strong spin‐lattice coupling provided by the high‐spin state of the Dy^3+^ ions as commonly in rare‐earth oxides.^[^ ^34^, ^35^, ^36^ ^]^ The alteration in the magnetic moment under an externally applied magnetic field induces energy variations, resulting in an opposite change in phonon angular momentum (lattice). Subsequently, this change is transferred to the lattice through spin‐lattice coupling, leading to a modification in dielectric permittivity. Additionally, MD anisotropy is in accordance with the magnetic anisotropy in each single crystal. Moreover, the weak but non‐negligible magnetic couplings between Dy^3+^ ions also influence the spin‐lattice interactions as well as magnetic anisotropy which yield a larger maximum value of Δ*ε*/*ε*(0 T) of **Dy3** SMM than the monuclear Dy‐SMM. Otherwise, although similar magnetic interactions between **Dy3** and the former ferroelectric Dy3, no prominent dielectric permittivity change under external magnetic field was observed in the ferroelectric one. Hence, molecular packing model is another crucial factor in SMMs because it will generate different spin‐lattice couplings.

## 3 Conclusion

In summary, we report the observation of MD effect in a triangular **Dy3** SMT exhibiting vortex magnetic moments and slow magnetic relaxations. Intrestingly, a negative MD effect is observed with a large anisortopy. Further investegations reveal that the MD effect is influenced by the strong spin‐lattice coupling of high spin state Dy^3+^ ions, the magnetic interactions between Dy^3+^ ions as well as molecular packing models in multinuclear clusters. Therefore, in addition to confirming the prediction made over the past decade, this work indicates that larger MD effect could be obtained in other multinuclear clusters and brings a new route for the future research on the design and synthesis of molecular multiferroics.

## 4 Experimental Section

Complex **Dy3** in this study has a similar synthesis method to the previous trinuclear Dy_3_ complex.^[^ ^12^ ^]^ It was synthesized by a solution method, Dy(NO_3_)_3_·6H_2_O (0.5 mmol, 228.3 mg) was slowly added to a 20 mL acetonitrile solution containing H_4_L (1.0 mmol, 273 mg) and LiOH (0.4 mmol, 16.8 mg). The mixture was moved to a vial after 2 h of stirring. In two days could obtain colorless crystals under the condition of 100°C heating and the yield is ≈60% (based on Dy). Single‐crystal X‐ray diffraction was performed on an Agilent SuperNova diffractometer (126 K) equipped and a Bruker D8 Venture diffractometer (30 K) equipped with graphite monochromated Mo‐Kα radiation (*λ* = 0.71073 Å). The crystal structure was solved by direct methods and refined by the full‐matrix least‐squares method on F^2^ with anisotropic thermal parameters for all nonhydrogen atoms using the SHELXL program. And the hydrogen atoms were located geometrically and refined isotropically (see Table S1, Supporting Information). Thermogravimetric analysis (TGA) was performed on a Netzsch TG 209 TG‐DTA analyser from 40 to 800°C under a nitrogen atmosphere with a heating rate of 10°C min^−1^. Powder X‐ray diffraction patterns were measured on a Rigaku Ultima IV diffractometer using Cu‐Kα radiation shown in Figure S2 (Supporting Information). The magnetism of the polycrystal sample and single crystal sample were measured on a Quantum Design superconducting quantum interference device magnetometer (MPMS XL‐7). Complete‐active‐space self‐consistent field (CASSCF) calculations on individual Dy^3+^ fragments of trinuclear complex based on X‐ray determined geometries at 30 K have been carried out with MOLCAS 8.4 and SINGLE_ANISO programs. The magnetodielectric permittivity of single crystal samples with an Andeen Hagerling 2700 capacitance bridge were performed on a Quantum Design Physical Property Measurement System (PPMS).

## Conflict of Interest

The authors declare no conflict of interest.

## Supporting information

Supporting Information

## Data Availability

The data that support the findings of this study are available from the corresponding author upon reasonable request.
