# Supplementary material for: Magnetodielectric Effect in a Triangular Dysprosium Single‐Molecule Toroics
Source: Adv Sci (Weinh). 2024 Jan 17;11(10):2308220. doi: 10.1002/advs.202308220 (PMC10933626; doi:10.1002/advs.202308220)
Supplement: Supplementary file 1 — Supporting Information [file ADVS-11-2308220-s001.pdf]

ADVANCED SCIENCE
Open Access

Supporting Information

for *Adv. Sci.*, DOI 10.1002/advs.202308220

Magnetodielectric Effect in a Triangular Dysprosium Single-Molecule Toroics

Yu-Xia Wang, Yicheng Zhou, Yinina Ma, Peipei Lu, Yi-Quan Zhang, Young Sun and Peng Cheng**

Supporting Information

Magnetodielectric Effect in a Triangular Dysprosium Single-Molecule Toroic

Yu-Xia Wang, Yicheng Zhou, Yinina Ma, Peipei Lu, Yi-Quan Zhang, Young Sun, and Peng Cheng**

Table of Contents

1. **Computational details**
page 3
2. Results and Discussion
Figure S1 Thermogravimetric curve of **Dy3**
page 4
Figure S2 Powder XRD patterns of **Dy3**
page 4
Figures S3 and **S4** Additional molecular structures
page 5
Figure S5 Calculated model structures of individual Dy^{3+} fragments in **Dy3**
page 5
Figure S6 The best fit results with the modified general Debye function for **Dy3**
page 6
Figure S7 The best fit to piecewise power law equations
page 7
Figure S8 The crystal orientation data for **Dy3**
page 8
Figure S9 Magnetic anisotropy of the single crystal sample for **Dy3**
page 9
Figure S10 Dielectricity of the single crystal sample for **Dy3**
page 10

Table S1 Crystallographic parameters of **Dy3**
page 11
Table S2 CSM calculations for three Dy sites in **Dy3**
page 12
Table S3 Fitted exchange couplings calculation
page 13
Table S4 Exchange energies calculation
page 14
Table S5 Calculated energy levels, *g* tensors and predominant m_J values
page 15
Table S6 Wave functions with definite projection of the total moment
page 16
Table S7 Angles between the main magnetic axes
page 17

References
page 17

1. Computational details

Trinuclear complex **Dy3** has three types of magnetic center Dy^{3+} ions indicated as Dy1, Dy2 and Dy3. Complete-active-space self-consistent field (CASSCF) calculations on individual Dy^{3+} fragments for complex **Dy3** (see Figure S5) on the basis of single-crystal X-ray determined geometry have been carried out with OpenMolcas[2] program package. Each of individual Dy^{3+} fragments in **Dy3** was calculated keeping the experimentally determined structures of the corresponding compound while replacing the other Dy^{3+} ions with diamagnetic Lu^{3+}.

The basis sets for all atoms are atomic natural orbitals from the ANO-RCC library: ANO-RCC-VTZP for Dy^{3+}; VTZ for close O and N; VDZ for distant atoms. The calculations employ the second order Douglas-Kroll-Hess Hamiltonian, where scalar relativistic contractions are taken into account in the basis set and the spin-orbit couplings are handled separately in the restricted active space state interaction (RASSI-SO) procedure.[3,4] Active electrons in 7 active orbitals include all *f* electrons (CAS (9 in 7for Dy^{3+})) in the CASSCF calculation. To exclude all the doubts, we calculate all the roots in the active space. We have mixed the maximum number of spin-free state which is possible with our hardware (all from 21 sextets, 128 from

224 quadruplets, 130 from 490 doublets) for each fragment. SINGLE_ANISO[5-7] program is used to obtain the energy levels, ***g*** tensors, magnetic axes, *et. al.* based on the above CASSCF/RASSI-SO calculations.

To fit the exchange interactions in complex **Dy3**, we took two steps to obtain them. Firstly, we calculated individual Dy^{3+} fragments using CASSCF/RASSI-SO to obtain the corresponding magnetic properties. Then, the exchange interaction between the magnetic centers was considered within the Lines model,[8] while the account of the dipole-dipole magnetic coupling was treated exactly. The lines model is effective and has been successfully used widely in the research field of *d* and *f*-elements single-molecule magnets.[9,10]

For **Dy3**, there are three types of $\tilde{J}$ and the Ising exchange Hamiltonian is:

$$\hat{H}_{exch} = -\tilde{J}_1\hat{\tilde{S}}_{Dy1}\hat{\tilde{S}}_{Dy2} - \tilde{J}_2\hat{\tilde{S}}_{Dy2}\hat{\tilde{S}}_{Dy3} - \tilde{J}_3\hat{\tilde{S}}_{Dy1}\hat{\tilde{S}}_{Dy3} \qquad \text{(S1)}$$

The $\tilde{J}_1 = 25\cos\varphi\, J_1$, where φ is the angle between the anisotropy axes on sites Dy1 and Dy2, and J_1 is the Lines exchange coupling parameter. The other two coupling constants of $\tilde{J}_2$ and $\tilde{J}_3$ have the similar expressions. The $\tilde{S}_{Dy}$ = 1/2 is the ground pseudospin on the Dy^{3+} site. $\tilde{J}_{total}$ is the parameter of the total magnetic coupling constant ($\tilde{J}_{total} = \tilde{J}_{dipolar} + \tilde{J}_{exchange}$) between magnetic center ions. The dipolar coupling can be calculated exactly, while the exchange coupling constant was fitted through comparison of the computed and measured magnetic susceptibilities using POLY_ANISO program.[5-7]

2. Results and Discussion

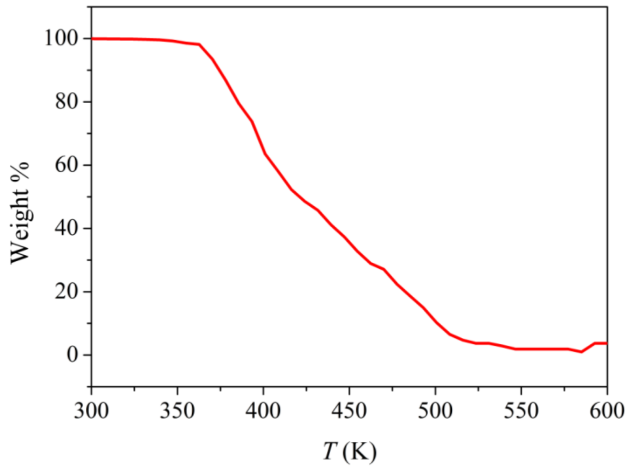

Figure S1 The thermogravimetric curve of **Dy3**.

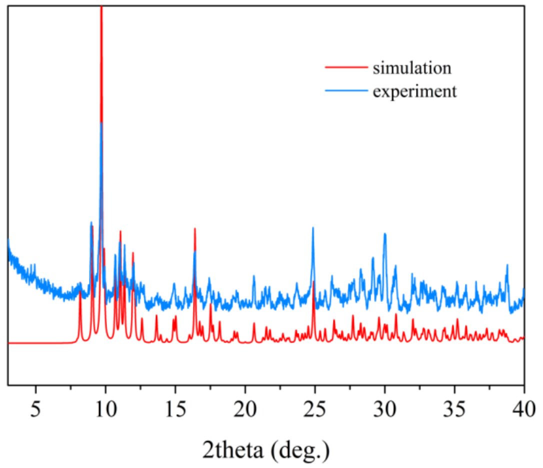

Figure S2 The powder XRD patterns of **Dy3**.

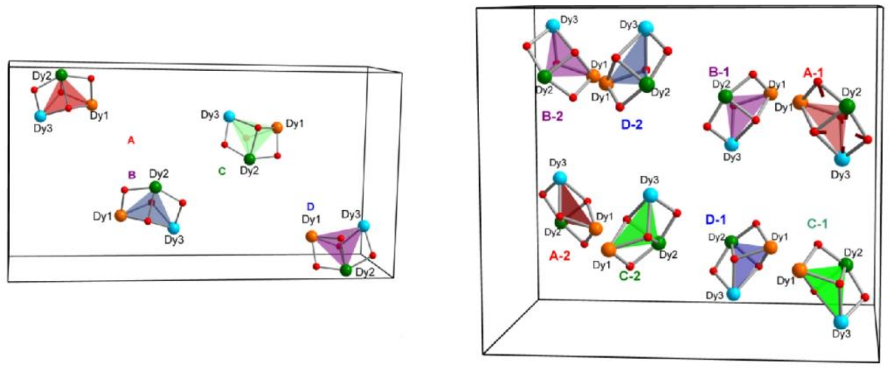

(a) **(b)**

Figure S3 The different oriented Dy3 triangular clusters per unit cell for ferroelectric Dy3 SMM[1] (a) and **Dy3** (b).

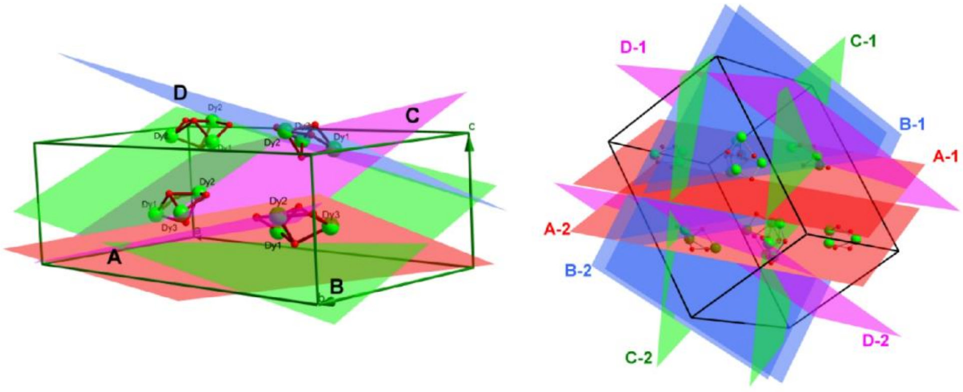

(**a**) (**b**)

Figure S4 The interlaced plans for each triangular per unit represented with different colors for ferroelectric Dy3 SMM[1] (a) and **Dy3** (b). Different triangular planes distinct each other by different colors.

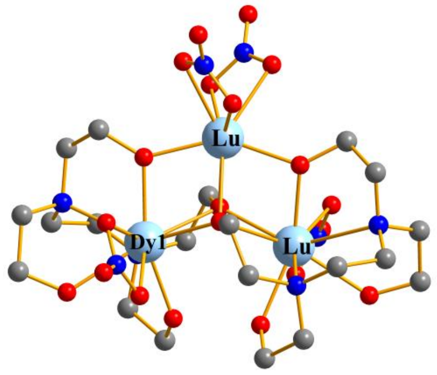

Dy1

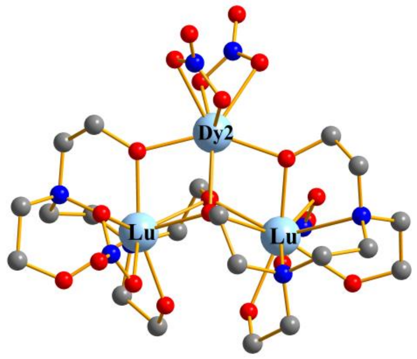

Dy2

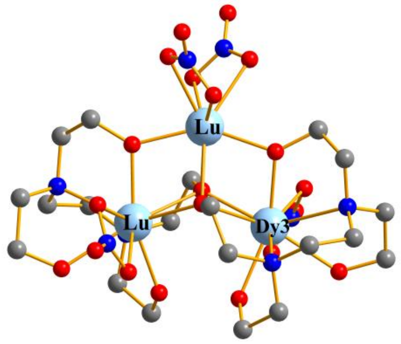

Dy3

Figure S5 Calculated model structures of individual Dy^{3+} fragments in complex **Dy3**; H atoms are omitted for clarify.

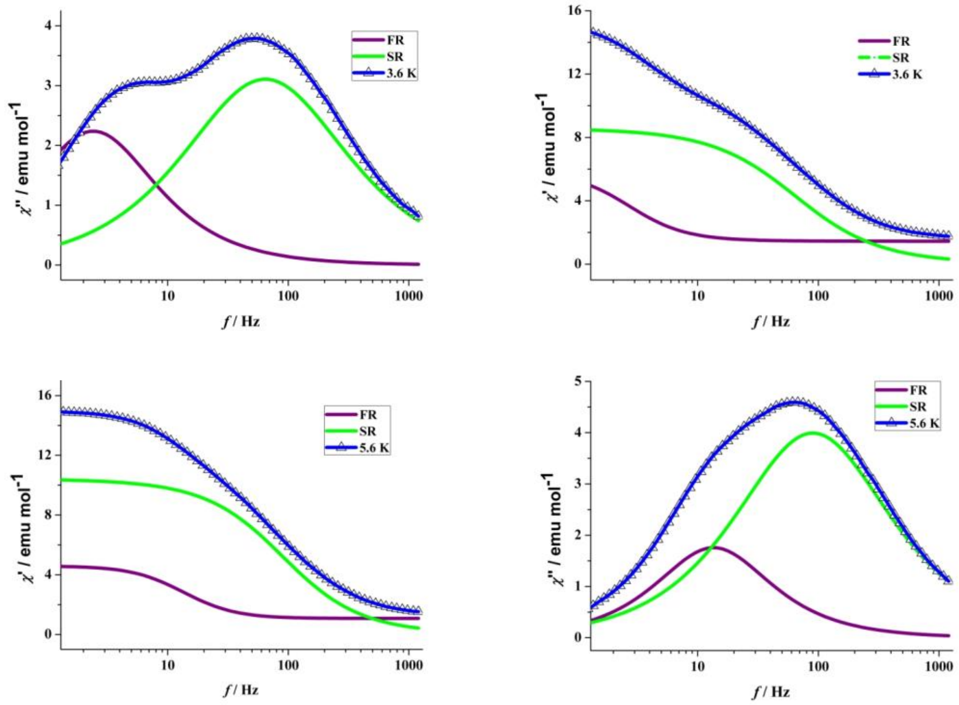

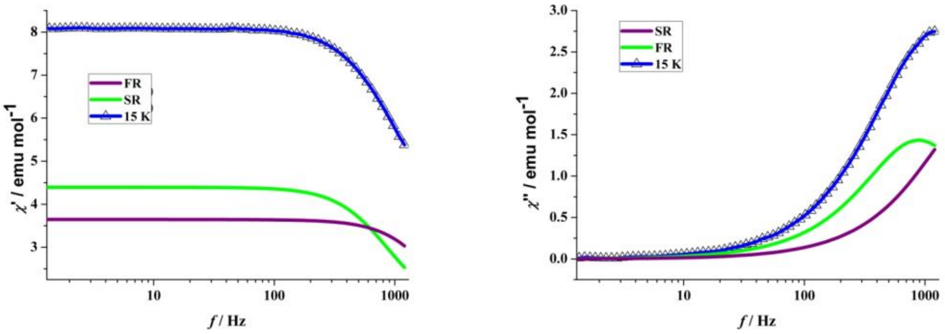

Figure S6 The best fit results with the modified general Debye function for **Dy3** at the temperatures between 3.6 K and 15 K, respectively.

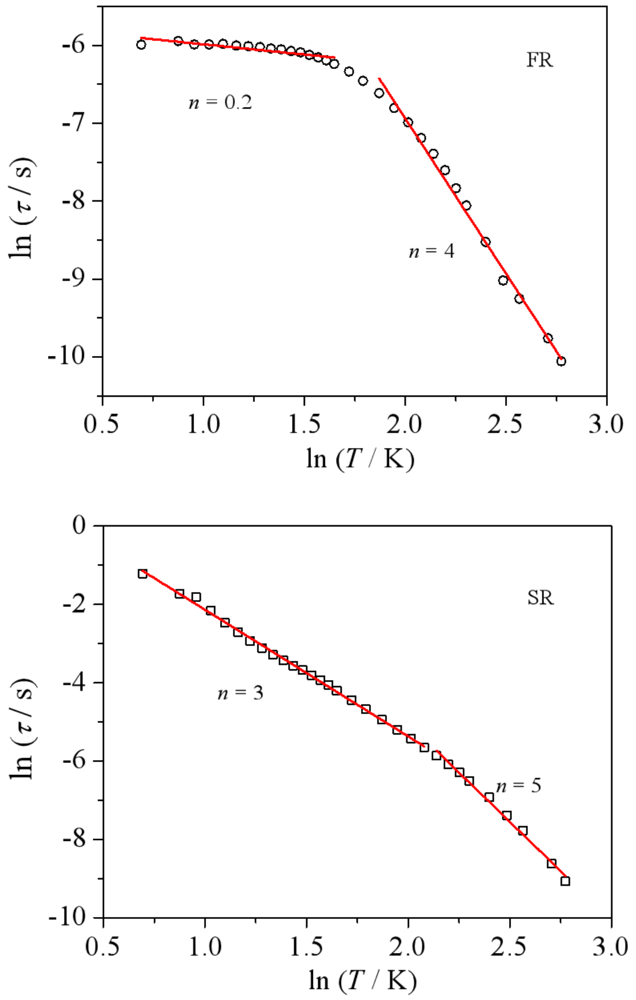

Figure S7 The solid red lines represent the best fit to piecewise power law equations.

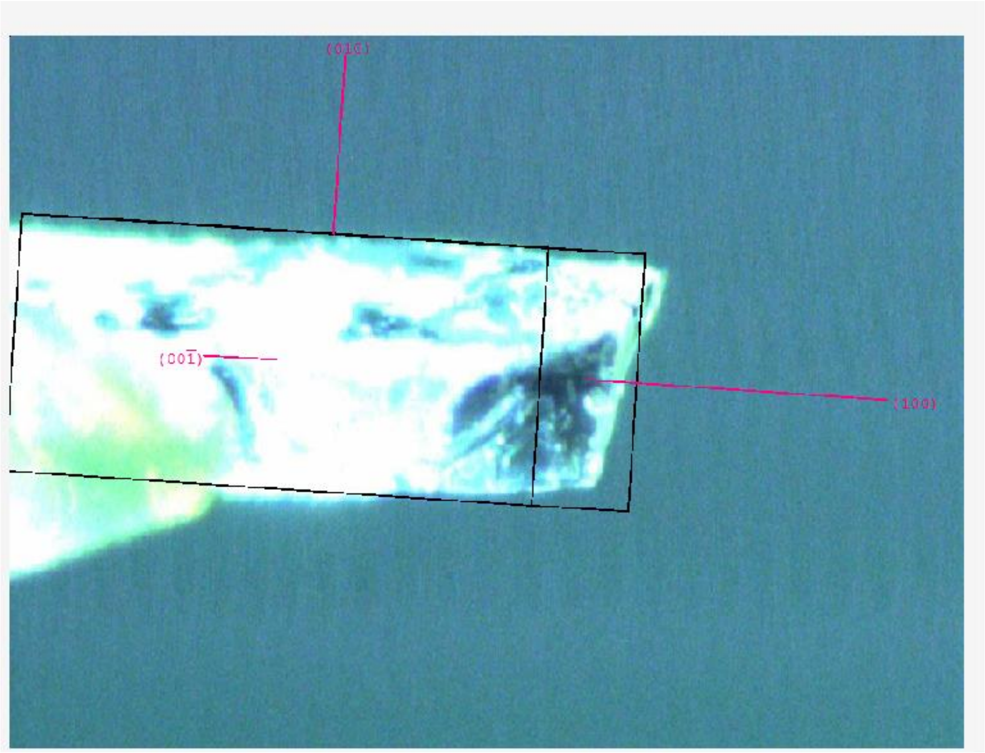

Figure S8 The crystal orientation of the single crystal of **Dy3**. The larger crystal face of (00$\bar{1}$) is named *ab* plane for clarity.

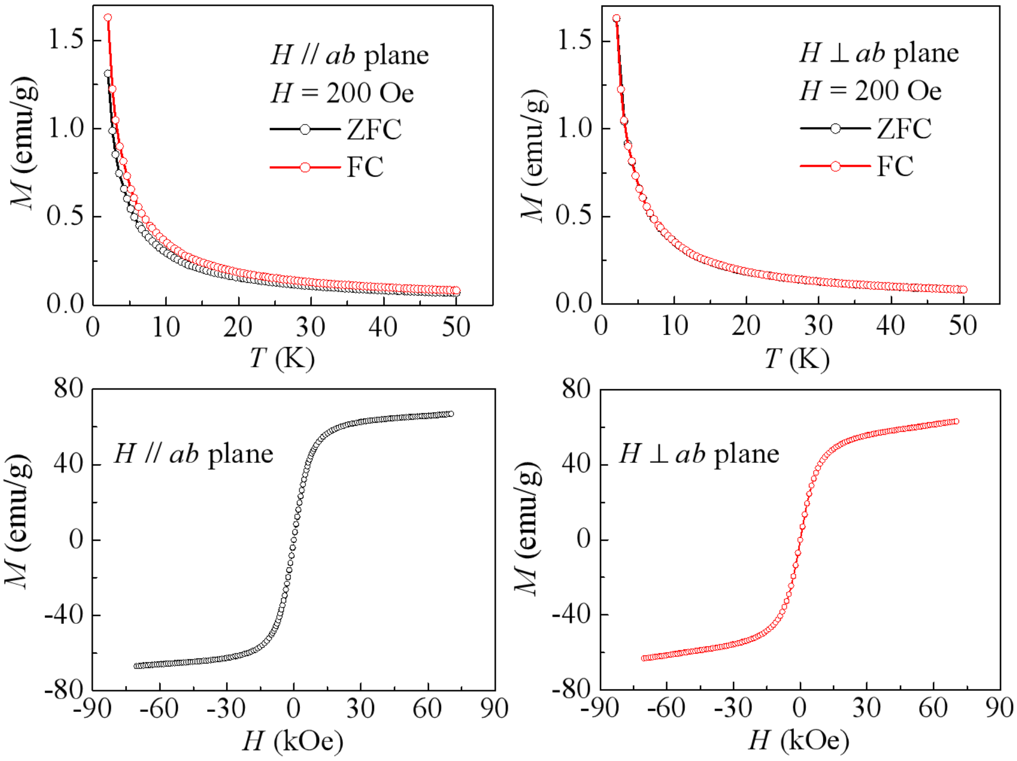

Figure S9 Magnetic anisotropy of the single crystal sample for **Dy3**. Zero-field cooling and field cooling curves and magnetic hysteric loops with *H* // *ab* plane and *H* ⊥ *ab* plane.

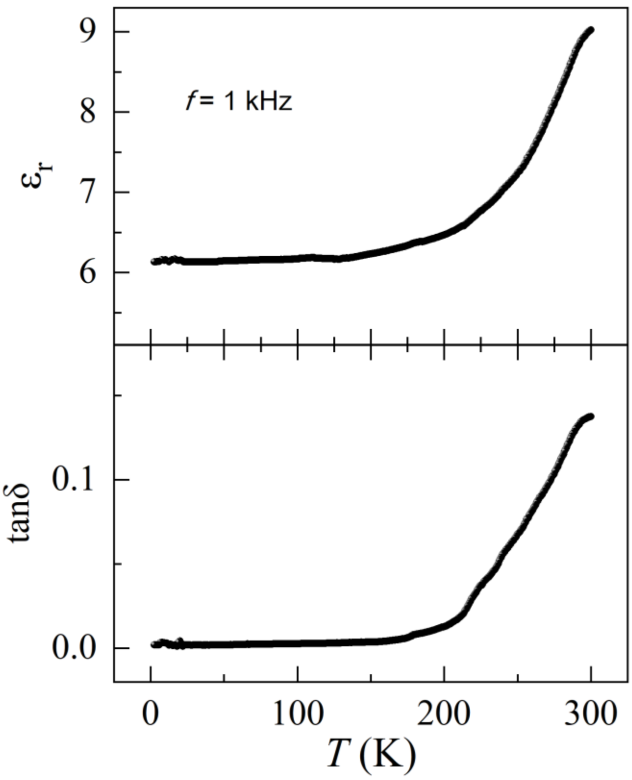

Figure S10 Dielectricity and the tangent loss of the single crystal sample for **Dy3** in the temperature range of 2–300 K. No dielectric anormalies indicates that there is no structure phase transation.

Table S1 The crystallographic parameters of **Dy3**.

CCDC	2304398	2270337
Formula	$C_{20}H_{43}Dy_3N_8O_{20}$	$C_{20}H_{43}Dy_3N_8O_{20}$
Formula weight	1203.12	1203.12
Temperature (K)	30(2)	126(2)
Crystal System	Orthorhombic	Orthorhombic
Space group	*Pbca*	*Pbca*
a (Å)	15.8600(6)	15.9847(7)
b (Å)	19.3894(8)	19.510(3)
c (Å)	22.0751(8)	22.1718(10)

$\alpha = \beta = \gamma$ (deg)	90	90
V (Å^3)	6788.4(5)	6914.6(11)
Z	8	8
Dc (g·cm^{-3})	2.354	2.311
Limiting indices	-18 ≤ h ≤ 18<br>-23 ≤ k ≤ 20<br>-26 ≤ l ≤ 26	-18≤h≤19<br>-15≤k≤23<br>-26≤l≤16
μ (mm^{-1})	6.631	6.510
R_{int}	0.0567	0.0588
GOOF	1.209	1.078
R_1	0.0289	0.0409
wR_2	0.0566	0.0868
$\triangle\rho_{\text{max}}$ (e Å^{-3})	0.85	1.381
$\triangle\rho_{\text{min}}$ (eÅ-3)	-1.33	-1.873

$R_1 = \sum||Fo| - |Fc||/\sum|Fo|$; $wR_2 = [\sum w(Fo^2 - Fc^2)^2/\sum w(Fo^2)^2]^{1/2}$

Table S2 CSM calculations for three Dy sites in **Dy3**.

[ML$_9$]	CSAPR-9	MFF-9	TCTPR-9
Dy1/Spherical capped square antiprism	2.691	3.179	3.211
	$C_{4\text{v}}$	C_s	$D_{3\text{h}}$
[ML$_8$]	SAPR-8	BTPR-8	JBTP-8
Dy2/square antiprism	2.825	3.373	3.551
	$D_{4\text{d}}$	$C_{2\text{v}}$	$C_{2\text{v}}$
[ML$_9$]	CSAPR-9	MFF-9	JCSAPR-9
	2.282	2.633	2.956

Dy3/Spherical capped square antiprism	C_{4v}	C_s	C_{4v}

D_{4d} = Square antiprism (SAPR-8); C_{2v} = Biaugmented trigonal prism (BTPR-8); C_{2v} = Johnson-Elongated triangular bipyramid (J14) (JBTPR-8); C_{4v} = Capped square antiprism (CSAPR-9); C_{4v} = Capped square antiprism J10 (JCSAPR-9); C_s = Muffin (MFF-9); D_{3h} = Tricapped trigonal prism (TCTPR-9).

Table S3 Fitted exchange couplings $\tilde{J}_{exch}$, the calculated dipole-dipole interactions $\tilde{J}_{dip}$ and the total constants $\tilde{J}_{total}$ between magnetic center ions in **Dy3** (cm^{-1}). The intermolecular interaction zJ' of **Dy3** was fitted to −0.02 cm^{-1}.

	$\tilde{J}_{exch}$	$\tilde{J}_{dip}$	$\tilde{J}_{total}$
J_1	−0.58	−5.27	−5.85
J_2	−0.93	−5.39	−6.32
J_3	−2.27	−2.74	−5.01

Table S4 Exchange energies E (cm^{-1}), the transversal magnetic moments Δ_t (μB) and the main values of the g_z for the lowest four exchange doublets of complex **Dy3**.

	E	Δ_t	g_z
1	0.0	1.130×10^{-10}	11.689
2	5.4	3.722×10^{-8}	43.796
3	5.6	5.235×10^{-8}	42.951
4	6.1	7.171×10^{-9}	28.152

Table S5 Calculated energy levels (cm^{-1}), $\boldsymbol{g}$ (g_x, g_y, g_z) tensors and predominant m_J values of the lowest eight Kramers doublets (KDs) of individual Dy^{3+} fragments for complex **Dy3** using CASSCF/RASSI-SO with OpenMolcas.

KDs	1_Dy1			1_Dy2			1_Dy3		
	E	***g***	m_J	*E*	***g***	m_J	*E*	***g***	m_J
1	0.0	0.006<br>0.006<br>19.825	±15/2	0.0	0.000<br>0.001<br>19.848	±15/2	0.0	0.045<br>0.071<br>19.675	±15/2
2	200.8	0.106<br>0.120<br>17.085	±13/2	277.8	0.082<br>0.098<br>17.015	±13/2	110.9	0.390<br>0.553<br>16.606	±13/2
3	431.3	0.569<br>1.018<br>13.494	±11/2	487.2	1.487<br>4.491<br>11.077	±11/2	239.5	2.327<br>3.756<br>12.553	
4	555.7	2.767<br>5.000<br>11.916		532.8	1.148<br>3.225<br>13.912		307.5	1.499<br>3.936<br>9.416	
5	606.5	0.476<br>4.468<br>12.675		604.3	2.753<br>3.398<br>14.747		350.1	1.678<br>2.754<br>13.546	
6	651.1	0.108<br>4.988<br>10.173		621.3	3.368<br>5.024<br>11.546		396.8	2.727<br>3.614<br>12.067	
7	691.8	0.072<br>1.924<br>8.389		714.6	0.478<br>0.964<br>16.978		468.9	0.838<br>1.057<br>17.222	
8	751.0	1.502<br>5.990<br>13.247		901.0	0.062<br>0.096<br>19.525		557.4	0.251<br>0.482<br>18.329	

Table S6 Wave functions with definite projection of the total moment $|m_J>$ for the lowest eight KDs of individual Dy^{3+} fragments for complex **Dy3**.

	E/cm	wave functions
1_Dy1	0.0	99.2%|±15/2>

<table>
<tr><td></td><td>200.8</td><td>96.5%|±13/2></td></tr>
<tr><td></td><td>431.3</td><td>84.3%|±11/2>+5.6%|±9/2></td></tr>
<tr><td></td><td>555.7</td><td>28.4%|±3/2>+23.8%|±9/2>+22.7%|±1/2>+13.8%|±5/2></td></tr>
<tr><td></td><td>606.5</td><td>56.2%|±1/2>+16.8%|±3/2>+12.1%|±9/2>+5.8%|±5/2></td></tr>
<tr><td></td><td>651.1</td><td>36.3%|±9/2>+18.6%|±7/2>+15.8%|±5/2>+13.9%|±3/2>+12.3%|±1/2></td></tr>
<tr><td></td><td>691.8</td><td>37.1%|±3/2>+28.6%|±5/2>+17.7%|±7/2>+9.4%|±9/2></td></tr>
<tr><td></td><td>751.0</td><td>48.1%|±7/2>+34.5%|±5/2>+11.9%|±9/2></td></tr>
<tr><td rowspan="8">1_Dy2</td><td>0.0</td><td>99.6%|±15/2></td></tr>
<tr><td>277.8</td><td>96.7%|±13/2></td></tr>
<tr><td>487.2</td><td>68.8%|±11/2>+13%|±1/2>+7.7%|±3/2></td></tr>
<tr><td>532.8</td><td>42.5%|±1/2>+21.5%|±3/2>+16.8%|±11/2>+10.3%|±5/2></td></tr>
<tr><td>604.3</td><td>26.3%|±5/2>+25%|±7/2>+23.3%|±3/2>+13%|±9/2></td></tr>
<tr><td>621.3</td><td>42.9%|±9/2>+16.1%|±7/2>+16%|±3/2>+12.5%|±5/2>+10.1%|±1/2></td></tr>
<tr><td>714.6</td><td>31%|±7/2>+29.3%|±5/2>+15.3%|±9/2>+13.5%|±3/2></td></tr>
<tr><td>901.0</td><td>23.7%|±7/2>+19.9%|±5/2>+17.4%|±3/2>+16.9%|±1/2>+16.3%|±9/2></td></tr>
<tr><td rowspan="8">1_Dy3</td><td>0.0</td><td>97.4%|±15/2></td></tr>
<tr><td>110.9</td><td>88.4%|±13/2>+3.6%|±11/2></td></tr>
<tr><td>239.5</td><td>30.9%|±11/2>+28.4%|±1/2>+14.3%|±5/2>+13.1%|±3/2></td></tr>
<tr><td>307.5</td><td>43.4%|±11/2>+21.7%|±3/2>+15.4%|±1/2>+5.4%|±13/2>+5%|±9/2></td></tr>
<tr><td>350.1</td><td>43.8%|±1/2>+20.8%|±3/2>+11.6%|±7/2>+10.9%|±9/2>+8.3%|±5/2></td></tr>
<tr><td>396.8</td><td>29.9%|±3/2>+27.4%|±5/2>+23.7%|±9/2>+9.1%|±1/2></td></tr>
<tr><td>468.9</td><td>34.2%|±7/2>+25.6%|±9/2>+24.9%|±5/2>+6.8%|±3/2></td></tr>
<tr><td>557.4</td><td>38%|±7/2>+25.6%|±9/2>+21%|±5/2>+6.6%|±11/2></td></tr>
</table>

Table S7 Angles between the main magnetic axes on Dy^{3+} ions in their ground KDs and the angles between the magnetic axes and the Dy_3 plane for **Dy3.**

Dy^{3+} sites	Dy1	Dy2	Dy3	Dy_3 plane
Dy1	0°	103.4°	155.3°	9.0°

Dy2	103.4°	0°	100.8°	4.6°
Dy3	155.3°	100.8°	0°	−5.9°

References

[1] (a) Y.-X. Wang, W. Shi, H. Li, Y. Song, L. Fang, Y. Lan, A. K. Powell, W. Wernsdorfer, L. Ungur, L. F. Chibotaru, M. Shen, P. Cheng, *Chem. Sci.* **2012**, *3*, 3366–3370. (b) Y.-X. Wang, Y. Ma, J.-S. Wang, Y. Yang, Y.-N. Guo, Y.-Q. Zhang, K.-J. Jin, Y. Sun, P. Cheng, *Adv. Sci.* **2022**, *9*, 2202979.

[2] I. F. Galván, M. Vacher, A. Alavi, C. Angeli, F. Aquilante, J. Autschbach, J. J. Bao, S. I. Bokarev, N. A. Bogdanov, R. K. Carlson, L. F. Chibotaru, J. Creutzberg, N. Dattani, M. G. Delcey, S. S. Dong, A. Dreuw, L. Freitag, L. M. Frutos, L. Gagliardi, F. Gendron, A. Giussani, L. González, G. Grell, M. Guo, C. E. Hoyer, M. Johansson, S. Keller, S. Knecht, G. Kovačević, E. Källman, G. Li Manni, M. Lundberg, Y. Ma, S. Mai, J. P. Malhado, P. Malmqvist, P. Marquetand, S. A. Mewes, J. Norell, M. Olivucci, M. Oppel, Q. M. Phung, K. Pierloot, F. Plasser, M. Reiher, A. M. Sand, I. Schapiro, P. Sharma, C. J. Stein, L. K. Sørensen, D. G. Truhlar, M. Ugandi, L. Ungur, A. Valentini, S. Vancoillie, V. Veryazov, O. Weser, T. A. Wesołowski, P.-O. Widmark, S. Wouters, A. Zech, J. P. Zobel, R. Lindh, *J. Chem. Theory Comput.* **2019**, *15*, 5925–5964.

[3] P. Å. Malmqvist, B. O. Roos, B. Schimmelpfennig, *Chem. Phys. Lett.* **2002**, *357*, 230–240.

[4] B. A. Heß, C. M. Marian, U. Wahlgren, O. Gropen, *Chem. Phys. Lett.* **1996**, *251*, 365–371.

[5] L. F. Chibotaru, L. Ungur, A. Soncini, *Angew. Chem.* **2008**, *120*, 4194–4197; *Angew. Chem. Int. Ed.* **2008**, *47*, 4126–4129.

[6] L. Ungur, W. Van den Heuvel, L. F. Chibotaru, *New J. Chem.* **2009**, *33*, 1224–1230.

[7] L. F. Chibotaru, L. Ungur, C. Aronica, H. Elmoll, G. Pilet, D. Luneau, *J. Am. Chem. Soc.* **2008**, *130*, 12445–12455.

[8] M. E. Lines, *J. Chem. Phys.* **1971**, *55*, 2977–2984.

[9] K. C. Mondal, A. Sundt, Y. Lan, G. E. Kostakis, O. Waldmann, L. Ungur, L. F. Chibotaru, C. E. Anson, A. K. Powell, *Angew. Chem. Int. Ed.* **2012**, *51*, 7550−7554.

[10] S. K. Langley, D. P. Wielechowski, V. Vieru, N. F. Chilton, B. Moubaraki, B. F. Abrahams, L. F. Chibotaru, K. S. Murray, *Angew. Chem. Int. Ed.* **2013**, *52*, 12014–12019.
